# A Potential Valorization Strategy of Wine Industry by-Products and Their Application in Cosmetics—Case Study: Grape Pomace and Grapeseed

**DOI:** 10.3390/molecules27030969

**Published:** 2022-01-31

**Authors:** Sara M. Ferreira, Lúcia Santos

**Affiliations:** 1 LEPABE—Laboratory for Process Engineering, Environment, Biotechnology and Energy, Faculty of Engineering, University of Porto, Rua Dr. Roberto Frias, 4200-465 Porto, Portugal; up201604659@fe.up.pt; 2 ALiCE—Associate Laboratory in Chemical Engineering, Faculty of Engineering, University of Porto, Rua Dr. Roberto Frias, 4200-465 Porto, Portugal

**Keywords:** by-products, bioactive compounds, grape pomace, grapeseed, antioxidants, cosmetics

## Abstract

Grape pomace and grapeseed are agro-industrial by-products, whose inadequate treatment generates socioeconomic and environmental concerns. Nevertheless, it is possible to valorize them by extracting their bioactive compounds, such as antioxidants (phenolic compounds), vitamin E and fatty acids. The bioactive compounds were extracted using solid-liquid extraction. The yields for phenolic compounds were 18.4 ± 0.4% for grape pomace, and 17.4 ± 0.4%, for grapeseed. For the oil, the yields were 13.3 ± 0.2% and 14.5 ± 0.3% for grape pomace and grapeseed. Antioxidant capacity was assessed by the assay with 2,2-diphenyl-1-picrylhydrazyl (DPPH), and showed that phenolic extract has higher antioxidant capacity than the oils. Grape pomace and grapeseed extracts exhibit, correspondingly, values of 90.8 ± 0.8 and 87.5 ± 0.5 of DPPH inhibition and IC_50_ of 48.9 ± 0.5 and 55.9 ± 0.7 μg_extract_·mL_DPPH_^−1^. The antimicrobial capacity was assessed by the disk diffusion test, and revealed that, phenolic extracts inhibit the growth of *Staphylococcus aureus* and *Staphylococcus epidermidis*. The obtained extracts were incorporated in 10 face cream formulations, with slight modifications in quantities of formulation stabilizers. Their stability was studied for 35 days, and this revealed the possibility of incorporating extracts and oils obtained from by-products as antioxidants in cosmetics, and replacing synthetic ones. As a future recommendation, microencapsulation of the extracts should be performed, in order to increase their stability.

## 1. Introduction

The agro-food industry has grown exponentially over the past years due to an increased need to satisfy customer needs. The main objective of this industry is to transform raw materials into food goods to facilitate consumption, making them safer at the same time. The rise in food production is one of the causes for the high amount of agro-industrial waste and by-products—such as pomace, peels, and seeds—generated [1]. The incorrect disposal and treatment of these residues have impacted the social, economic, and environmental levels. For instance, at the environmental level, these residues contribute to an increase in the emission of gases with greenhouse effect [2]. The greater awareness of the population towards this problem created the concern in giving a new life to these residues and by-products from different sectors [3]. Indeed, some of the waste produced has nutritional and functional potential, as well as biological properties, and have been classified as by-products [4]. Hereupon, allying the existing characteristics of by-products with the ideology of sustainability and the circular economy model, there is a massive preoccupation to find new opportunities to reuse, recycle and give a new life to by-products [5,6]. The majority of by-products are rich in bioactive compounds (BAC), for example, phenolic compounds (PC), vitamins, dietary fibers, unsaturated fatty acids, flavonoids. These compounds can be extracted and applied in numerous applications in different industries, such as the cosmetic and pharmaceutical industry, food and feed, agriculture, and so forth [7]. Furthermore, reusing BAC represents a measure to protect the environment, at the same time that helps to create and develop value-added products [8].

**Figure 1:**
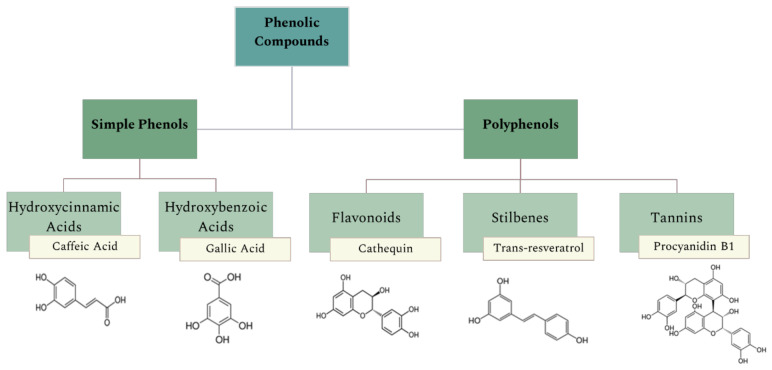
Different categories of phenolic compounds and examples of each class.

Grapes are one of the most valued crops in the world. In fact, the annual production worldwide exceeds 60 thousand tons. Near 80% of grapes are used for wine production, which generates high amount of organic and inorganic residues, residual water, and greenhouse gases [9,10,11]. Grape pomace (GP) is the main residue associated with this activity, and it is characterized as a solid material divided into two fractions: seedless pomace—composed of pulp, skin, and stem—and grapeseed (GS). GP is generated after pressing and fermentation steps and represents about 20% of the original weight of the grape [1]. GP composition may vary since the proportion in each phase can be different, as the seed fraction is between 38–52% [9]. This by-product is rich in BAC, such as unsaturated fatty acids, dietary fibers, vitamins, and natural antioxidants, mainly in the form of PC (as phenolic acids, flavonoids, and proanthocyanins) [9,12,13]. PC are plants’ secondary metabolites, of which the quantity available on the plant depends on multiple factors as cultivation technique, growing conditions, harvest process, processing, and storage. In terms of their chemical structure, PC exhibit at least one aromatic ring and have one or more hydroxyl groups. As shown in Figure 1, PC are divided into two categories: simple phenols and polyphenols [12,14].

Depending on the extraction technique, GP extracts are characterized for showing significant amount of flavonoids, specifically flavan-3-ols and cyanidin, it also exhibits phenolic acids, namely hydroxybenzoic, and hydroxycinnamic acids, and lastly, it possesses stilbenes and tannins. Around 60–70% of total extractable PC from grapes are contained in GS, being flavonoids (such as catechin and epicatechin), phenolic acids, and procyanidins the most usual ones. These compounds are very appealing to different industries due to their vast set of biological properties. Studies have proven that these compounds exhibit antimicrobial, antiviral, antiaging, antiallergic, and anticancer properties; nevertheless, their antioxidant capacity is the one that stands out the most. PC can act as natural antioxidant since they can neutralize oxidative stress, by inhibiting free radical production, interrupting chain auto-oxidation reactions, and so forth [15,16].

On the other hand, GP and GS can also be used as raw materials to obtain oils by using non-polar extraction solvents. In fact, the oils obtained from GP and GS are mainly composed of unsaturated fatty acids, like linoleic acid (the main fatty acid present, around 70–75%), oleic, and palmitic acid—relevant for the cosmetic industry due to their emollient properties. These oils have a low concentration of linolenic acid, which is an advantage (mainly in cosmetic formulations), since in high concentrations, it can create unpleasant smells and reduce oil stability. Vitamin E is also present in GP and GS oils, displaying a high amount of tocopherols (α, β, γ e δ) and tocotrienols (α, β, γ e δ) [17]. This vitamin presents very relevant characteristics for many industries, such as the cosmetic and food industry [18].

**Figure 2:**
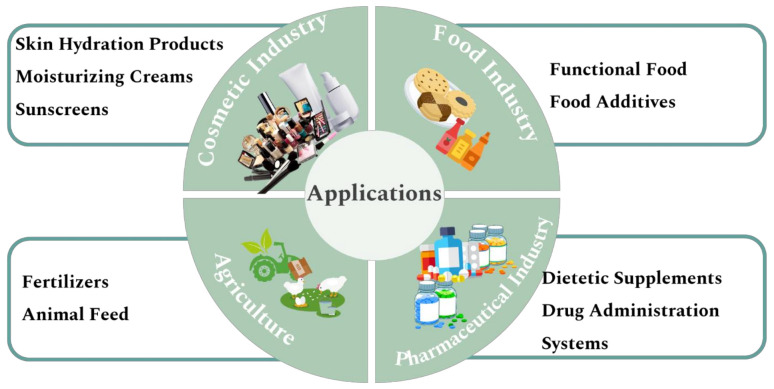
Miscellaneous applications of grape pomace and grapeseed extracts and oils in various industries.

According to the literature, GP and GS extracts and oils are used in many diverse industries—for instance, the food, and cosmetic industry—with very different purposes, as shown in Figure 2. The extracts from GP and GS are rich in bioactive compounds that have advantages since they exhibit therapeutical and functional properties, beneficial to the human being [19,20,21]. Some recent studies, exhibited in Table 1, show that extracts obtained from these by-products have been incorporated in both industries. However, the research and investigation on this topic is mainly focused on the incorporation and fortification of the extracts into foods.

Over the past years, the cosmetic industry has evolved to create and develop new products, entitled green cosmetics, that is, products in which formulations are of natural origin or mainly with natural ingredients. These ingredients have advantages against synthetic compounds since they are biodegradable and exhibit therapeutic and functional properties, beneficial to the human being [20,21]. This industry has focused on using BAC from natural sources—such as agricultural by-products, plants, amid others —not only as it helps to develop a value-added product (since the product is more sustainable) but also generates a more appealing products for consumers [3,21].

Personal appearance is a requirement of great importance, leading the current population to look for ways to achieve it. One of the strategies adopted to accomplish these goals is the use of cosmetic products. The most common cosmetics are moisturizing creams. These are an emulsion of immiscible liquid phases—an aqueous and oily phase—where one of the liquids is dispersed on the other in the form of small droplets. The base formulation of face moisturizers consists in a solvent, emollients, humectants, emulsifiers, neutralizers, and thickening agents. Formulation stabilizers – such as chelating agents, antioxidants, and preservatives, and aesthetical attributes, for example fragrances, dyes, and pigments, can also be added to the formulation to make it more stable and attractive to consumers [28,29,30].

The presence of antioxidant agents is crucial to the performance and maintenance of face cream since these molecules are capable of avoiding oxidation of the product since many ingredients present on the formulation are susceptible to suffer oxidation, protecting the face cream. Therefore, these substances are used to increase cosmetics’ shelf life and as a bioactive ingredient are beneficial to the skin as they protect against free radicals, which lead to skin aging, loss of elasticity, and wrinkles [31]. Butylhydroxytoluene (BHT) is a synthetic antioxidant used extensively in cosmetic formulations. Emerging studies revealed that synthetic antioxidants are associated with negative impacts on human health, for example allergic reactions, and carcinogenic, and toxic consequences [32]. Fortunately, nowadays, the pursuit of cosmetics with ingredients from natural sources has been growing, enhancing this industry improvement [33,34]. Thus, there has been a tendency to incorporate antioxidants of natural origin in cosmetic products. Hence, the opportunity to take advantage of the antioxidant properties presented by the PC and oils present in the GP and GS arises since they can be incorporated in moisturizing creams, thus increasing the product’s commercial value.

Fragrances are important ingredients to be present in cosmetic formulations, as they help to disguise unpleasant smells and odors of certain ingredients present in the formulation, and moreover, they also confer a specific aroma to make it more pleasing and alluring for consumers. Essential oils (OE) are often used as fragrances in cosmetics due to their pleasant smell and as they can be obtained from natural sources, such as peels, flowers, and seeds from plants [35,36]. The global production of oranges is in excess of 300 thousand tons a year. The consumption and use of this fruit as a raw material generates high quantities of waste; around 50 to 60% per fruit, with the skin being the main wastage [37]. Fortunately, orange peels have OE in their composition that can be extracted and used in the cosmetic industry (perfumes, deodorants, soaps, creams), pharmaceutical industry (antimicrobial and anti-inflammatory agents), and food industry (flavoring agents and preservatives) [38,39]. For this reason, orange peels were used to obtain EO to incorporate as fragrances in the developed cream.

The purpose of this work was to create and develop a face moisturizing cream, mainly composed of natural ingredients, and increase its value, by the addition of PC extract and oils from GP and GS, by-products from the winery industry. This way, it is possible to valorize agricultural by-products while creating a more appealing product, increasingly sought after by consumers. PC extracts were used as antioxidant ingredients to prevent the oxidation of fatty compounds present in the formulation, increasing the stability of the face cream. At the same time, GP and GS oils also present advantages in being incorporated in the face cream since they can act as emollients due to the presence of fatty acids in their composition. The present work pretends to show that extracts and oils obtained from by-products with bioactive properties can replace synthetic ingredients in cosmetics.

## 2. Results and Discussion

### 2.1. Extraction of Extracts and Oils from Grapeseed and Grape Pomace

Phenolic compounds are relatively polar compounds; therefore, alcoholic solvents are favored for their extraction. Ethanol is considered one of the most suitable solvents for the extraction of PC, not only as it is recognized as GRAS but also due to its low boiling point, which decreases energy costs and simplifies the process of elimination of solvent from the sample. The extraction yield for the phenolic compounds from GP was 18.4 ± 0.4% and for the extraction using GS as raw material was 17.4 ± 0.4%.

Regarding the extraction of the oil from GS and GP, n-hexane was selected as an extraction solvent due to its nonpolar nature. It is one of the most used solvents due to its nonpolar characteristics, high stability, and relatively low boiling point. The achieved yields were 13.3 ± 0.2% and 14.5 ± 0.3% for GP oil and GS oil, respectively.

The obtained values are within the literature values span [40].

### 2.2. Antioxidant Capacity

To ascertain the antioxidant capacity of phenolic extract and oil from GP and GS, the essay with DPPH was the chosen method. The results were expressed in DPPH inhibition percentage and in the necessary concentration of the sample to inhibit 50% of DPPH (IC_50_). IC_50_ value was determined from the calibration curve of the percentage of DPPH inhibition in function of sample concentration. Table 2 exhibits the obtained results.

The results presented in Table 2 reveal that the antioxidant capacity of phenolic extracts is particularly higher than the values obtained for the oils. In effect, the results were expected since the extracts are rich in PC (such as catechins, anthocyanins, among others), which are an antioxidant capacity-enhancing factor. In fact, these compounds can act as reducing agents, hydrogen donors, metal chelators, and superoxide radical scavengers [41]. These properties are associated with their chemical structure since the antioxidant capacity relates to their capacity to reduce or inhibit free radicals by transferring a hydrogen atom from the hydroxyl group, linked to the aromatic ring [42]. In the case of the oils, antioxidant capacity is due to the presence of tocopherols and tocotrienols [43].

Comparing the obtained values with those in the literature [44,45,46], it is possible to affirm that the values are similar to those reported. The slight distinction of the values can be associated with the variety of grape used, maturation phase, cultivation conditions, extraction conditions, among other variables. The obtained results show that antioxidant capacity is higher for GP extract than for GS extract. This result can be explained by the GP constitution since it is composed not only of seeds but also of skins. Red grapes’ skin is rich in trans-resveratrol, and this PC has a high antioxidant capacity, therefore this can be the main cause for the increase in antioxidant capacity in GP extract [47,48,49]. The obtained results prove that both GS extract and GP extract display high antioxidant capacity, since the value of IC_50_ for both samples is between 50 and 100 µg_extract_∙mL_DPPH_^−1^, which validates the possibility of using them as antioxidant agents from natural sources [50].

Considering GS oil, the results shown in Table 2, demonstrate that this oil has a faintly higher antioxidant capacity than GP oil. The attained results are accordant with those in the literature. The difference between the values for GS and GP oil can be a result of the tocopherol and tocotrienols concentration present on the extracts [51]. Regardless of the low antioxidant capacity of GS and GP oils, the achieved results indicate that it is possible to use extracts and oils from winemaking industry by-products, as antioxidant ingredients. Besides, they can act as emollient agents, a class of compounds often used in the cosmetic industry, in these cases with the advantage of being of natural origin, which makes the products more appealing to consumers.

### 2.3. Antimicrobial Capacity

The antimicrobial capacity of GP and GS extract and oil was evaluated using the Kirby–Bauer disc diffusion test, studied against *E. coli* (Gram-negative bacteria)*, S. aureus,* and *S. epidermidis* (Gram-positive bacteria). The average of the diameters of the inhibition halos (in mm) corresponding to the tested samples is revealed in Table 3.

Both GP and GS extract exhibit activity against Gram-positive bacteria, however, there was no inhibitory effect against *E. coli* [52]. These results were expected since it is reported that polyphenolic extracts are more effective against Gram-positive bacteria. Gram-negative microorganisms have a unique cell structure, with a double phospholipidic layer, which is responsible for the low susceptibility of these bacteria due to the repulsion between PC and the lipopolysaccharide present on the surface of the bacteria membrane [53]. According to the literature, bacteria from the Staphylococcus family, are the most susceptible to the action of PC, which is compatible with the obtained results [52,54]. GP extract proves to have a slightly more inhibitory effect on the microorganisms in the study, than GS extract. The results show that both extracts can have similar composition, and that they may be exploitable as antibacterial agents in cosmetics.

The antimicrobial capacity of GP and GS oils was not possible to measure since it did not display inhibition halo. Nevertheless, this does not mean that the oils are incapable of inhibiting the growth of microorganisms since the halo can be present under the disk. However, it was expected that the oils were capable of inhibiting the growth and development of Gram-positive microorganisms, due to the abundance of lipophilic components present in their composition and the presence of tocopherols and tocotrienols [55,56,57]. The obtained results prove that there is potential for GP and GS extracts to be used as preservatives in cosmetics formulations.

### 2.4. Stability Tests

After the production of the face moisturizing creams (FB and F1 to F9), their stability was evaluated. Therefore, the creams were subject to different stability tests for 35 days.

Sensory analysis was carried out to determine how the GP (grape pomace) and GS (grapeseed) extracts would affect the organoleptic properties of the creams. It was found that, the color and smell remained practically unchanged throughout the studying period. All formulations exhibit a beige coloration—apart from F6 and F7 that showed a pink color—and FB (base formulation) presented a slightly more yellowish tone than the others. Concerning the smell, all face creams preserved their orange scent, except for FB. The presence of antioxidant agents—present in F1 to F9—helps to avoid the oxidation of the ingredients of the formulation, and consequently prevent the development of unpleasant odors. Therewithal, all face creams kept their appearance, and this means they maintained their homogeneity without any phase separation and no granules were observed during the testing period. Therefore, it is possible to affirm that the emulsions were stable and did not experience changes in terms of sensorial analysis.

**Figure 3:**
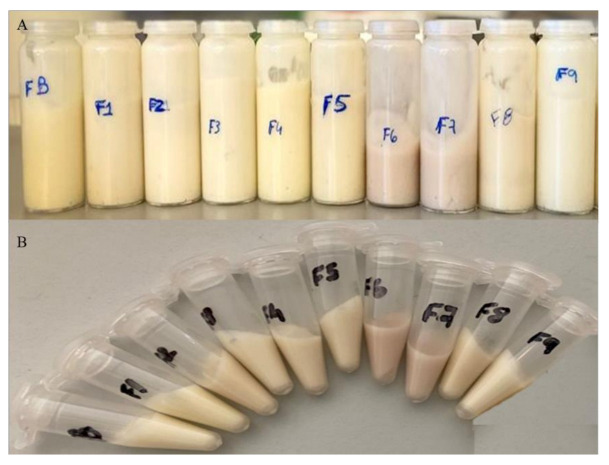
Face creams appearance after thermal stability test (**A**) and centrifugation test (**B**).

Accelerated stability assays were performed to evaluate the product’s performance, under extreme conditions. Figure 3 shows the obtained results after the centrifugation test and thermal stability. After the centrifugation essay, all the creams proved to have a stable emulsion, since none of them exhibit phase separation. Additionally, none of the essays evidenced phase separation after the thermal stability test. Even though there were slight weight variations, these were despised since the fluctuations were minimal (inferior to 0.5%). Furthermore, after the performance of the accelerated tests, all the organoleptic properties, appearance, and homogeneity of the samples were kept unaffected. Subsequently, it is possible to confirm that the emulsions of the 10 creams are stable, and that they are resistant to mechanical and temperature shocks.

**Figure 4:**
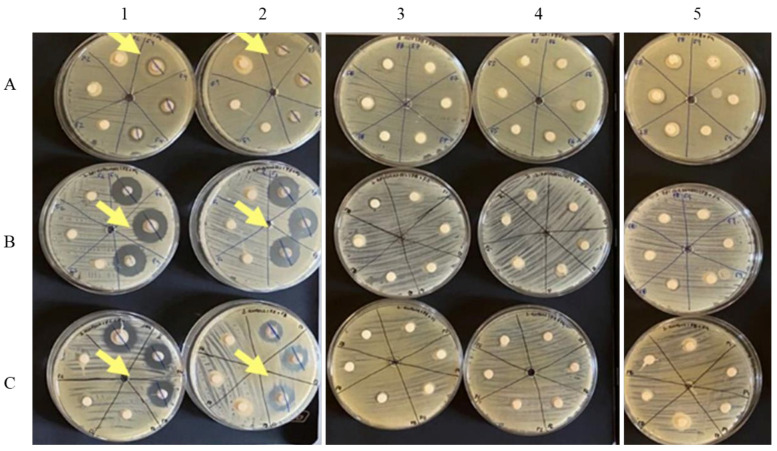
Results for the antimicrobial capacity test of the creams, against *S. aureus* (**A**), *S. epidermidis* (**B**) and *E. coli* (**C**). Legend: 1—Creams F1 and F6; 2—Creams F2 and F7; 3—Creams FB and F3; 4—Creams F4 and F5; 5—Creams F8 and F9.

Moreover, the antimicrobial capacity of the face creams was assessed by performing disk diffusion tests against *E. coli*, *S. aureus*, and *S. epidermidis*. The obtained results are shown in Figure 4 and Table 4. Of all the samples, only F6 and F7 revealed the capacity to inhibit the development and growth of microorganisms (displayed in Figure 4, Petri dishes A1, A2, B1, B2, C1and C2). This result can be related to the addition of betaine since this ingredient has the antimicrobial capacity, protecting the samples from contaminations [58]. All other results, including the sample with synthetic antioxidant, did not show an inhibitory effect.

**Figure 5:**
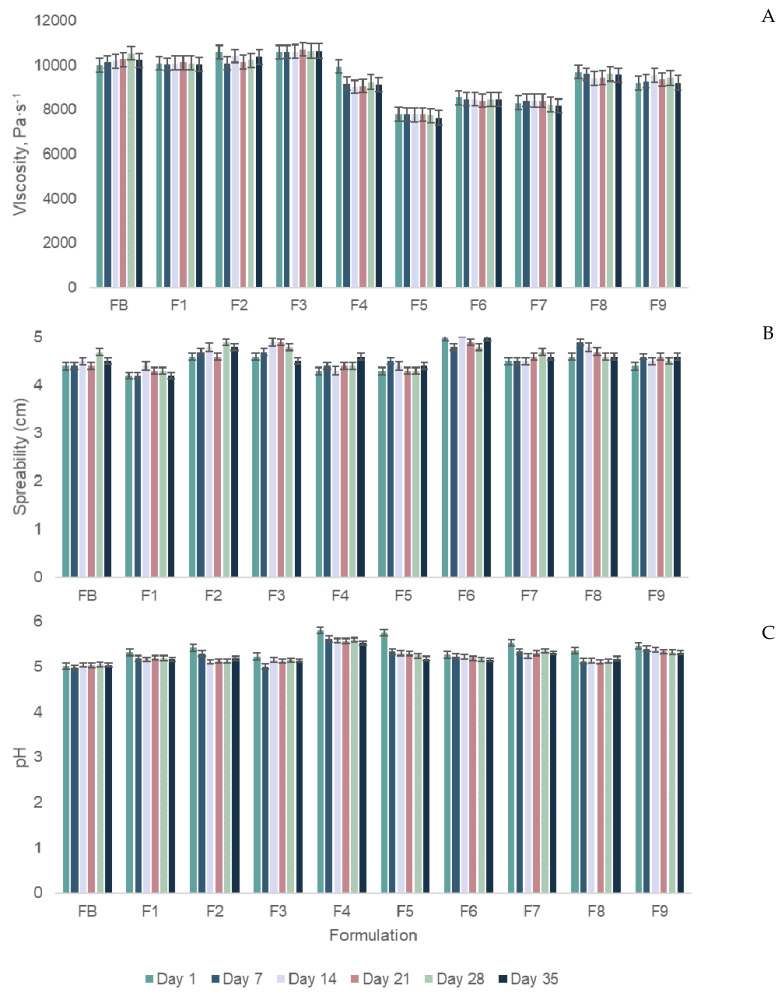
Oscillations of viscosity (**A**), spreadability (**B**) and pH (**C**) values, during 35 days, for the different samples.

Regarding the physical and chemical properties, such as viscosity, spreadability, and pH of the developed creams, Figure 5, displays the variations of these properties for 35 days.

From Figure 5, it is possible to verify that the oscillations in the viscosity and spreadability of the samples were minor. There were also small fluctuations in the pH value for the different essays. Nevertheless, the pH of healthy skin is in the range of 4.5 to 6.0, which means that face creams must be included in this span [59]. Therefore, even with the values fluctuations, the obtained pH values were kept in the desired range, which makes the samples compatible with the skin.

In order to assess if the produced face moisturizing creams were compatible with the human skin, the skin patch test was performed. For that, the test was initially performed with five people (three women and two men, between the ages of 21 to 62). After 24 h, none of the volunteers exhibited any type of adverse reaction, such as irritation, itchiness, redness, or inflammation after using the creams. Afterwards, the same study was performed with a more significant sample, constituted by 45 people. In this inquiry, 95.6% of the participants stated that no adverse reaction was detected after the application of the creams. Thereby, it is possible to confirm that the produced face creams are compatible with the skin.

The results prove that extracts of by-products of the winemaking industry, GP and GS, can be incorporated in cosmetic formulations, allowing formulations with identical functions, mainly antioxidant capacity, to those achieved in formulations with synthetic antioxidant. Hence, there is potential to valorize these secondary products extracting their BAC and incorporation in cosmetic formulation, creating value-added products.

### 2.5. Lipid Oxidation

**Figure 6:**
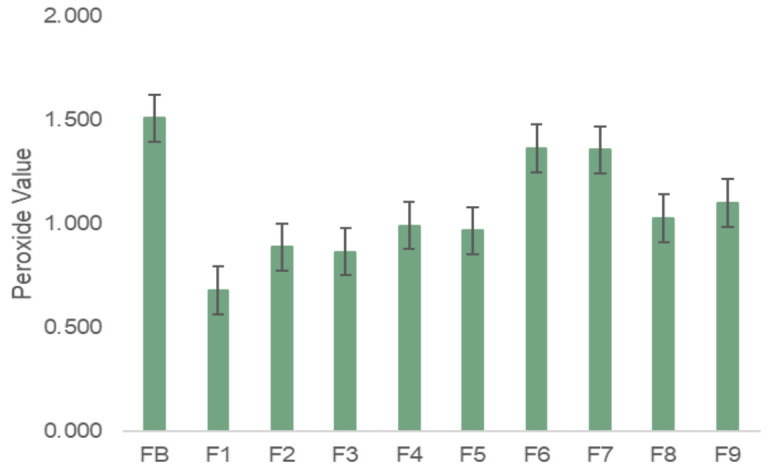
Peroxide value (PV), in milliequivalents of O_2_ per sample kilogram, for the evaluated formulations.

Face moisturizing creams contain ingredients susceptible to lipid oxidation. This is a frequent phenomenon, which occurs in formulations that have ingredients of a lipid nature that affect the final quality of the products. Lipid oxidation leads to the development of unpleasant odors, color changes, affects the shelf-time and performance of the product [60]. Due to the complexity of the analysis, there is no specific method to assess the degree of oxidation of creams. However, one of the most used tests in the cosmetic industry is the determination of the peroxide value (PV). This value helps determine the extent of oxidation of fats and oils since it quantifies the amount of peroxides present in the formulation. Since the substances are primary products of oxidation, their presence in the sample reveals that there was oxidation of the product [61]. Figure 6 presents the achieved results of PV (peroxide value) for the different samples.

The determination of the peroxide value aimed to compare the efficiency of the different extracts and oils in lipid oxidation, in comparison with the synthetic antioxidants.

A high PV means that the extension of products lipid oxidation was high [62]. From the analysis of Figure 6, it is possible to observe that FB (base formulation) is the essay with the highest PV (1.51 ± 0.2 mEq∙kg^−1^_sample_), and therefore, it is possible to conclude that, as anticipated, this sample exhibited a higher extent of lipid oxidation. This result was expected, since no antioxidant (antioxidant) agent was added and, consequently, the oxidation was not averted. Unavoidably, F1, which was used as standard, shows a lower PV (0.68 ± 0.3 mEq∙kg^−1^_sample_) due to BHT (synthetic antioxidant) addition, for this ingredient avoids and delays the oxidation of lipids, protecting the cream from deterioration. The presence of antioxidant in the formulation causes them to react with free radicals decreasing products’ oxidation. On the other hand, creams F2 and F3 —to which was added GS phenolic extract and GP phenolic extract, respectively—presented higher PV than F1. The peroxide value for F2 and F3 were, respectively, 0.89 ± 0.1 mEq∙kg^−1^_sample_ and 0.87 ± 0.6 mEq∙kg^−1^_sample_. Nevertheless, the values are 40% lower than FB (without the incorporation of antioxidant), which shows that phenolic extracts can prevent and delay oxidation. Essays F4 and F5, with addition of grapeseed oil reveal PV of 0.99 ± 0.5 mEq∙kg^−1^_sample_ and 0.97 ± 0.8 mEq∙kg^−1^_sample_, respectively. Essays F8 and F9, where grape pomace oil was added exhibited, correspondingly, PV of 1.03 ± 0.3 mEq∙kg^−1^_sample_ and 1.1 ± 0.4 mEq∙kg^−1^_sample_. It is possible to conclude that the addition of oils contributed to an increase in the PV, in comparison with F1. Given that GP and GS oils are rich in compounds of lipid nature that are fats, these compounds end up oxidizing, increasing lipid oxidation in the creams. This result was anticipated since these oils are rich in unsaturated fatty acids that are extremely vulnerable to oxidation, however, the PV values presented are well below that registered for FB (without the addition of an antioxidant). Essays F6 and F7 revealed the highest PV values (1.36 ± 0.2 mEq∙kg^−1^_sample_ and 1.34 ± 0.7 mEq∙kg^−1^_sample_, respectively) of the creams with the addition of formulation stabilizers. This result shows that the use of betaine, as the only emulsifier of the formulation, influences the oxidation of the creams, increasing the extension of this phenomenon. However, it is possible to verify that all essays register lower PV values than the value presented in FB, without the incorporation of extracts and oils.

The obtained results reveal that the formulation with the addition of BHT (F1) helps to prevent more lipid oxidation of the cream. Nevertheless, formulations F2 and F3 displayed a good performance avoiding the oxidation phenomenon. It is noteworthy that, BHT is a synthetic antioxidant, and thereby cosmetic regulation limits the quantity that can be applied in cosmetics, which is between 0.1–0.5% [63]. Since the phenolic extracts can also contain other bioactive compounds, such as vitamins, they can be added in higher quantities than BHT, which can increase the protection of the formulations from oxidation. On the other hand, microencapsulation could be a useful technique to be employed to the extracts, since it can help to protect them from degradation and oxidation.

Therefore, the obtained results reveal that phenolic extracts, obtained from winery by-products–grapeseed and grape pomace–have high antioxidant capacity and have the ability to protect creams from degradation and oxidation. It also shows that there is a possibility that these extracts can act as antioxidant agents and replace synthetic ingredients, such as BHT, a commercial antioxidant.

## 3. Materials and Methods

### 3.1. Reagents and Agro-Industrial by-Products

The extraction solvents ethanol (Ref. 1.02371.1000, C_2_H_6_O, CAS 64-17-5) and n-hexane (CH_3_(CH_2_)_4_CH_3_, CAS 110-54-3) were obtained from VWR (Fontenay-sous-Bois, France) and Valente e Ribeiro (Idanha, Portugal), respectively. For the antioxidant and antimicrobial capacity, DPPH (Ref. D9132, C_18_H_12_N_5_O_6_, CAS 1898-66-4), and ascorbic acid (Ref. PHR1008, C_8_H_8_O_6_, CAS 50-81-7) were used and purchased from Sigma Aldrich (St. Louis, MO, USA). For the face moisturizing creams the used reagents were glycerine (Ref. COSM-01216), xanthan gum (Ref. GOMA-00762), coconut oil (Ref. INCOCO-00185), and betaine (Ref. COR-COSM-00969) acquired from GranVelada (Zaragoza, Spain), soy lecithin (Ref.L0023, CAS 8002-43-5), purchased from TCI (Tokyo, Japan) and BHT (Ref.47168, C_15_H_24_O, CAS 128-37-0) purchased from Sigma Aldrich (St. Louis, MO, USA). For lipid oxidation tests, dehydrated barium chloride (Ref.217565, BaCl_2_.2H_2_O, CAS 10326-27-9) obtained from Sigma Aldrich (St. Louis, MO, USA), iron sulphate (II) (Ref. 24244.232, FeSO_4_∙7H_2_O, CAS 7782-63-0) and hydrochloric acid (Ref. 20255.290, HCl, CAS 7647-01-0) were bought in VWR (Fontenay-sous-Bois, France), ammonium thiocyanate (Ref.A10632, CH_4_N_2_S, CAS 1762-95-4) acquired from Alfa Aesar (Haverhill, MA, USA) and chloroform (Ref.438607, CH_3_Cl, CAS 67-66-3) and methanol (Ref. 414816, CH_3_OH, CAS 67-56-1) were purchased from Carlo Erba (Barcelona, Spain). A Merck Millipore Mill-Q water purification equipment, with 18.2 Ω of electric resistance (Billerica, MA, USA), used for deionised water. Grape pomace and grapeseed samples acquired from Alfândega da Fé, Bragança, Portugal, coming from three different grape castes: Touriga Nacional, Touriga Francesa and Tinta-roriz. Orange peels were obtained from oranges produced in Algarve, Portugal.

### 3.2. Methods

#### 3.2.1. Extraction of Phenolic Extract and Oil from Grapeseed and Grape Pomace

To extract the bioactive compounds from GS and GP it was necessary to perform a pre-treatment of raw materials. Thereby, GS and GP were washed three consecutive times with distillate water to remove any impurities present. Afterward, the samples were lyophilized at −90 °C and 1.3 Pa in a lyophilizer (SP Scientific, New York, USA). Finally, the seeds and pomace were ground to increase the surface area and passed through sieves of different mesh sizes. The medium size pieces (18 and 50 mesh) were chosen for the extraction step.

The main BAC present in GS and GP have different polarities. Therefore, ethanol was chosen as the solvent for the extraction of PC due to its polar characteristics, and n-hexane was chosen for the extraction of the oil since it is a non-polar solvent. Solid-liquid extraction, with a Soxhlet extractor, was the selected technique, where the extraction conditions were: 225 mL of solvent for 45 g of sample, solvent sample ratio of 5:1 (V/m), the extraction time was of 105 min, and the temperature was 80 °C and 70 °C, for the ethanolic extract and oil, respectively. Subsequently, the solvent was evaporated in a rotary evaporator (Bϋchi R-200, Flaiwil, Switzerland) with a bath temperature of 45 °C and 35 °C for the phenolic extract and oil, correspondingly. Furthermore, the samples were subjected to a constant stream of nitrogen to achieve total evaporation of the solvents. The extracts were then covered in foil paper, to prevent light degradation, and stored in a refrigerator until further use. The extractions were performed in triplicates and the yields were expressed as the mean and standard deviation of the three essays.

#### 3.2.2. Extraction of Essential Oil from Orange Peels

Initially the peels were washed with water to increase the contact area with the extraction solvent. The oil was obtained by hydrodistillation, and the conditions were 100 g of orange peels to 200 mL of distillate water, which gives a solvent sample ratio of 2:1 (V/m), for 1 h. To finalize the extraction, the oil was separated from the solvent using a micropipette to collect it. The achieved yield was 2.31% ± 0.11%, which is slightly lower than the values in the literature, which were around 3% [64]. The extractions were performed in triplicates and the yields were expressed as the mean and standard deviation of the three essays.

#### 3.2.3. Antioxidant Capacity

Antioxidant capacity was determined through the essay with 2,2-diphenyl-2-picrylhydrazyl radical (DPPH^•^) [65]. Initially, a 0.2 mM DPPH^•^ stock solution in methanol was prepared and a calibration curve was obtained considering multiple dilutions of the DPPH^•^ stock solution. To each well of the microplate, 100 μL of methanol was added followed by the addition of the same volume of sample. The content of each well was diluted eight times. Afterward, 100 μL of the 0.2 mM DPPH solution was added to each well of the plate. Finally, the microplate was covered with the lid to avoid any evaporation, wrapped in aluminum foil to protect the radicals from light degradation, and left to incubate for 30 min at room temperature. The absorbance was read using a microplate reader (Synergy, HT, Biotek, USA) at 515 nm. The inhibition percentage of DPPH (% I) was measured according to Equation (1).

(1) $$\%\;\mathrm{DPPH}\;\mathrm{Inhibition}=\left(A\_\left(\mathrm{control}-\right)\;A\_\mathrm{sample}\right)/A\_\mathrm{control}\;\times 100$$

#### 3.2.4. Antimicrobial Capacity

The antimicrobial capacity of all the extracts was assessed using the Kirby–Bauer method, commonly known as the disc diffusion test. The procedure was performed according to [66]. For that, solutions were prepared for both extracts and oils. Thereby, 1.5 g of the different samples were weighed and dissolved in 5 mL of aqueous solution, with 2% DMSO. The solutions were covered with foil paper and stored at 5 ºC, until further use. The culture medium used was the Plate Count Agar, PCA, and the target microorganisms were *Staphylococcus aureus* (335 PF), *Staphylococcus epidermidis* (DSM 20044-1115-001), and *Escherichia coli* (DSM 1103). The suspensions were prepared from a pure culture, in a solution with 0.9% of NaCl, whose turbidity was adjusted to 0.5 in the McFarland scale. The plates were inoculated with the strain. Then, sterile disks were added to the plate and 7 μL of sample and controls—with ultrapure water as negative control; 14% ascorbic acid solution as a positive control—were added. The plates were left to incubate at 37 °C for 24 h. Finally, the halos obtained on each disk were analyzed and compared to the control samples to evaluate the antimicrobial effects of each extract.

#### 3.2.5. Face Cream Moisturizer Production

To produce the face cream moisturizers ten different creams—one base formulation (FB) and nine formulations (F1 to F9)—were produced, to study the effect of the different extracts and oils on the base formulation. The process started by heating up to 70–75 °C, separately, phase A (aqueous phase)—composed of ultrapure water, glycerin, and xanthan gum—and phase B (oil phase)—containing coconut oil, sweet almond oil, soy lecithin, and betaine – using a thermostatic water bath (VWB2, VWR, Fontenay-sous-Bois, France). Subsequently, phase B was added to phase A, and proceeded to the homogenization of the mixture, with a high-performance homogenizer, Ultra-Turax (IKA T18 Digital ULTRA-TURRAX^®^, Staufen, Germany), during 2 min and a velocity of 12,000 rpm, maintaining the temperature. Afterward, the mixture was cooled down to 40°C, and the formulation stabilizers—such as BHT (synthetic antioxidant), GP and GS extract, and GP and GS oil—were added on the assays F1 to F9. Table 5 shows the differences between the 10 face creams. The quantities (%) of all used ingredients are similar to those reported in the literature and follow the legislation, namely the formulation stabilizers. However, the present formulation includes only the essential ingredients to create a face cream in order to interpret the effect of the adding the extracts/oils.

#### 3.2.6. Stability Tests

After the production of the different face cream moisturizers, stability tests were performed to assess the importance of the added formulation stabilizers (extracts and oils). All the tests, apart from the thermal stability test, were performed at room temperature (20–25 °C).

To study the stability of the produced face creams pH, viscosity, and spreadability of the samples were measured at t_1_ (the day of the production), and 7, 14, 21, 28, and 35 days after the production of the creams, to detect any alteration during this period. To assess the pH, the value was measured directly from the sample, this means, without sample dissolution, with a digital pH meter (XS Instruments, Capri, Italia) [67]. For the measurement of the viscosity of the samples, a viscometer (ViscoStar plus, Fungilab, New York, USA) was used [67]. For that, the viscometer spindle was inserted into a glass flask, containing 15 g of the samples, and left there for 10 min, to stabilize the viscosity value. The spreadability of the face creams was determined weighing 0.15 g of sample was placed between two glass plates, then, a 45.0 g weight was placed, carefully, on top. After 1 min, the weight was removed and the diameter of the spread sample was measured with a ruler [68].

Skin compatibility was assessed by the skin patch test. A small amount of cream was placed on the interior part of the arm, previously washed, on which a patch was placed. The cream was considered incompatible, if there was any sign of irritation, redness, and itchiness, after 24 h [69].

To verify the stability of the emulsion, a centrifugation test was performed. The test was carried out with a centrifuge (Centrifuge 5424, Eppendorf, Hamburg, Germany) at the speed of 5000 rpm, for 10 min, at 25 °C [67].

Thermal stability was studied resorting to the method of Freeze-thaw cycles. The samples were placed in bottles and were subjected to four cycles, 24 h each, between a freezer (4 ± 3 °C), room temperature (20–25 °C), a stove (40 ± 0.5 °C) and room temperature (20–25 °C) [67].

#### 3.2.7. Lipid Oxidation

Lipid oxidation tests were executed following a literature protocol using the Peroxide Value (PV) [70]. Initially, 0.1 g of sample were mixed in 9.8 mL of a 7:3 (*v*/*v*) solution of chloroform–methanol, and the final mixture was vortexed for 2 to 4 s. Afterward, 50 µL of an ammonium thiocyanate solution was added, and vortexed for 2 to 4 s, followed by 50 µL of an iron (II) solution. The samples were incubated for 5 min at room temperature, and the absorbance was measured using a UV-Vis spectrophotometer (V-530, Jasco, OK, USA) at 500 nm.

## 4. Conclusions

This study aimed to evaluate the possibility of wine industry by-products, grape pomace, and grapeseed, being used to obtain bioactive extracts and oils, and their incorporation in cosmetics, assessing the odds of replacing synthetic antioxidants. The solid–liquid extraction yields of the phenolic extracts and oils were good and in accordance with the literature. It was proved that both the extracts and oils exhibited antioxidant capacity. However, the phenolic extracts displayed higher antioxidant power due to the presence of phenolic compounds in their composition. Phenolic extracts also demonstrated antimicrobial capacity, inhibiting the growth of Gram-positive microorganisms, such as *Staphylococcus aureus* and *Staphylococcus epidermidis*. Finally, the stability tests and lipid oxidation assay allowed us to conclude that the incorporation of extracts and oils from GP (grape pomace) and GS (grapeseed) in cosmetics have similar results to cosmetics with synthetic antioxidants, such as BHT. Therefore, it is possible to incorporate extracts and oils prevenient from wine industry by-products in cosmetic formulations, allowing the development of a sustainable cosmetic product while fulfilling the circular economy cycle. Phenolic compounds tend to be oxidaze and degraded. To avoid this effect, it would be desirable to microencapsulate the obtained extracts and then compare the stability of the creams with the incorporation of extracts and microparticles with extract.

## Figures and Tables

**Table 1:** Studies regarding the incorporation of grape pomace and grapeseed extracts in the food and cosmetics field.

	Objectives	Results	Reference
**Food Industry**	Application of the grape skin and seeds extract, rich in antioxidants in Petit Suisse cheese and evaluation of its technological, sensorial, and functional properties.	No difference was detected, neither in sensorial acceptance nor in total phenolic content in fortified cheese	[22]
	Protein and phenolic characterization and evaluation of antioxidant properties of goat’s milk powder enriched with grapeseed extract	The addition of grapeseed extract enhanced the antioxidant capacity of the milk.	[23]
	Development of a functional food ingredient by incorporation of grape pomace extract.	Higher bioactive content and better physical properties.	[24]
**Cosmetic Industry**	Evaluation of the potential use of grape pomace extract in personal-care products	The extract presented appropriate characteristics to be incorporated in cosmetics and good acceptability by consumers	[25]
	Comparison between a cosmetic serum with phytosomes of grapeseed extract and a serum containing grapeseed extract and evaluation of phenolic penetration in skin	The incorporation of phytosomes from grapeseed extract can enhance the penetration of phenolic compounds into the skin	[26]
	Incorporation of phenolic extract from grape pomace in sunscreen, and evaluation of their antioxidant, and their photostability, and in vitro sun protection factor	The antioxidant activity was higher in formulations with the extract. The alliance between the extract and UV filters increased the sun protection factor of 81%.	[27]

**Table 2:** DPPH inhibition percentage and IC_50_ values for phenolic extract and oil from GP and GS.

	DPPH (% DPPH Inhibition)	DPPH (IC_50_) (μg_sample_·mL_DPPH_^−^^1^)
Phenolic Extract GP	90.8 ± 0.8	48.9 ± 0.5
Phenolic Extract GS	87.5 ± 0.5	55.9 ± 0.7
GP Oil	33.7 ± 0.2	296.1 ± 0.9
GS Oil	34.6 ± 0.2	283.3 ± 0.9

GP—Grape pomace; GS—Grapeseed.

**Table 3:** Inhibition halos (diameter values in mm), after 24 h of incubation.

Microorganism	d_halo_ (mm)			
	Phenolic Extract GP	Phenolic Extract GS	GP Oil	GS Oil
*S. aureus*	12.7 ± 0.9	12.0 ± 0.0	ND	ND
*S. epidermidis*	14.3 ± 0.5	12.2 ± 0.2	ND	ND
*E. coli*	ND	ND	ND	ND

ND—Not detected: it was not possible to measure the inhibition halo diameter being small. GP: Grape Pomace; GS: Grapeseed.

**Table 4:** Inhibition halos (diameter values in mm), after 24 h of incubation, for the different face creams.

Microorganism.	d_halo_ (mm)									
	FB	F1	F2	F3	F4	F5	F6	F7	F8	F9
*S. aureus*	ND	ND	ND	ND	ND	ND	18.3 ± 0.9	20.7 ± 1.7	ND	ND
*S. epidermidis*	ND	ND	ND	ND	ND	ND	23.0 ± 0.8	23.7 ± 0.9	ND	ND
*E. coli*	ND	ND	ND	ND	ND	ND	9.7 ± 0.9	12.7 ± 0.9	ND	ND

ND—Not detected: it was not possible to measure the inhibition halo diameter being small.

**Table 5:** Composition (in mass percentages) of the different formulations developed.

Ingredients		FB	F1	F2	F3	F4	F5	F6	F7	F8	F9
		(%)									
Phase A	Ultrapure Water	74.0	73.9	73.9	73.9	73.6	73.6	73.6	73.6	73.6	73.6
	Glycerin	7.6	7.6	7.6	7.6	7.6	7.6	7.6	7.6	7.6	7.6
	Xanthan Gum	0.6	0.6	0.6	0.6	0.6	0.6	0.6	0.6	0.6	0.6
Phase B	Coconut Oil	7.6	7.6	7.6	7.6	7.6	7.6	7.6	7.6	7.6	7.6
	Sweet Almond Oil	6.4	6.4	6.4	6.4	6.4	6.4	6.4	6.4	6.4	6.4
	Soy Lecithin	3.0	3.0	3.0	3.0	3.0	3.0	-	-	3.0	3.0
	Betaine	0.8	0.8	0.8	0.8	0.8	0.8	3.8	3.8	0.8	0.8
Formulation Stabilizers	BHT	-	0.1	-	-	-	-	-	-	-	-
	Phenolic Extract GS	-	-	0.1	-	0.1	-	0.1	-	0.1	-
	Phenolic Extract GP	-	-	-	0.1	-	0.1	-	0.1	-	0.1
	GS Oil	-	-	-	-	0.1	0.1	0.1	0.1	-	-
	GP Oil	-	-	-	-	-	-	-	-	0.1	0.1
	Orange EO	-	-	-	-	0.2	0.2	0.2	0.2	0.2	0.2

FB—Base formulation; F—Formulation with slight modifications in relation to FB; - Not present; BHT—synthetic antioxidant; GS—Grapeseed; GP—Grape pomace; EO—Essential oil.

## Data Availability

Not applicable.

## Sample Availability

Samples of the compounds, grape pomace and grapeseed, are available from the authors.

## Abbreviations

BAC	Bioactive Compounds
EO	Essential Oil
GP	Grape Pomace
GS	Grapeseed
PC	Phenolic Compounds
PV	Peroxide Value
